# Exercise priming with transcranial direct current stimulation: a study protocol for a randomized, parallel-design, sham-controlled trial in mild cognitive impairment and Alzheimer’s disease

**DOI:** 10.1186/s12877-021-02636-6

**Published:** 2021-12-04

**Authors:** Celina S. Liu, Nathan Herrmann, Bing Xin Song, Joycelyn Ba, Damien Gallagher, Paul I. Oh, Susan Marzolini, Tarek K. Rajji, Jocelyn Charles, Purti Papneja, Mark J. Rapoport, Ana C. Andreazza, Danielle Vieira, Alex Kiss, Krista L. Lanctôt

**Affiliations:** 1 Department of Pharmacology & Toxicology, University of Toronto, 1 King’s College Circle, Toronto, ON M5S 3K1 Canada; 2 Neuropsychopharmacology Research Group, Hurvitz Brain Sciences Program, Sunnybrook Research Institute, 2075 Bayview Avenue, Toronto, ON M4N 3M5 Canada; 3 Department of Psychiatry, University of Toronto, Toronto, ON Canada; 4 Department of Psychiatry, Division of Geriatric Psychiatry, Sunnybrook Health Sciences Centre, 2075 Bayview Avenue, Toronto, Ontario M4N 3M5 Canada; 5 Department of Biology, Faculty of Science, The University of Western Ontario, London, ON Canada; 6 Cardiovascular Prevention and Rehabilitation Program, KITE - Toronto Rehabilitation Institute, University Health Network, 347 Rumsey Road, Toronto, ON M5G 1R7 Canada; 7 Adult Neurodevelopment and Geriatric Psychiatry Division, Centre for Addiction & Mental Health, 80 Workman Way, Toronto, ON M6J 1H4 Canada; 8 Toronto Dementia Research Alliance, University of Toronto, Toronto, ON Canada; 9 Family & Community Medicine, Sunnybrook Health Sciences Centre, 2075 Bayview Avenue, Toronto, ON M4N 3M5 Canada; 10 Institute for Clinical Evaluative Sciences, Sunnybrook Health Sciences Centre, Toronto, ON Canada

**Keywords:** Transcranial direct current stimulation, Exercise, Priming, Mild cognitive impairment, Alzheimer’s disease, Neurogenesis, Angiogenesis, Cognition, Neuropsychiatric symptoms, Clinical trial

## Abstract

**Background:**

Transcranial direct current stimulation (tDCS) is a non-invasive type of brain stimulation that uses electrical currents to modulate neuronal activity. A small number of studies have investigated the effects of tDCS on cognition in patients with Mild Cognitive Impairment (MCI) and Alzheimer’s disease (AD), and have demonstrated variable effects. Emerging evidence suggests that tDCS is most effective when applied to active brain circuits. Aerobic exercise is known to increase cortical excitability and improve brain network connectivity. Exercise may therefore be an effective, yet previously unexplored primer for tDCS to improve cognition in MCI and mild AD.

**Methods:**

Participants with MCI or AD will be randomized to receive 10 sessions over 2 weeks of either exercise primed tDCS, exercise primed sham tDCS, or tDCS alone in a blinded, parallel-design trial. Those randomized to an exercise intervention will receive individualized 30-min aerobic exercise prescriptions to achieve a moderate-intensity dosage, equivalent to the ventilatory anaerobic threshold determined by cardiopulmonary assessment, to sufficiently increase cortical excitability. The tDCS protocol consists of 20 min sessions at 2 mA, 5 times per week for 2 weeks applied through 35 cm^2^ bitemporal electrodes. Our primary aim is to assess the efficacy of exercise primed tDCS for improving global cognition using the Montreal Cognitive Assessment (MoCA). Our secondary aims are to evaluate the efficacy of exercise primed tDCS for improving specific cognitive domains using various cognitive tests (n-back, Word Recall and Word Recognition Tasks from the Alzheimer’s Disease Assessment Scale-Cognitive subscale) and neuropsychiatric symptoms (Neuropsychiatric Inventory). We will also explore whether exercise primed tDCS is associated with an increase in markers of neurogenesis, oxidative stress and angiogenesis, and if changes in these markers are correlated with cognitive improvement.

**Discussion:**

We describe a novel clinical trial to investigate the effects of exercise priming before tDCS in patients with MCI or mild AD. This proof-of-concept study may identify a previously unexplored, non-invasive, non-pharmacological combination intervention that improves cognitive symptoms in patients. Findings from this study may also identify potential mechanistic actions of tDCS in MCI and mild AD.

**Trial registration:**

Clinicaltrials.gov, NCT03670615. Registered on September 13, 2018.

## Background

Dementia is a neurocognitive disorder (NCD) characterized by a progressive deterioration of cognitive and functional abilities [1]. Alzheimer’s Disease (AD) is the most common form of dementia, accounting for approximately 60-70% of all cases [2]. The diagnosis of a NCD, according to the Diagnostic and Statistical Manual of Mental Disorders -5th edition (DSM-5), can be classified as mild or major. Mild NCD, often referred to as Mild Cognitive Impairment (MCI), requires the presence of modest cognitive deficits without interference in everyday activities, whereas major NCD including AD requires both significant cognitive deficits and a reduced ability to perform everyday activities [3].

Cognitive problems are core deficits in MCI and AD. The earliest and most common clinical manifestation involves episodic memory impairment and a diminished ability to encode new material into long-term memory [4]. The temporal lobes of the brain are key structures in episodic memory, learning, and recall, and are damaged in AD [5]. These deficits are closely associated with neuronal hypoactivity and synaptic dysfunction [6–10].

Cholinesterase inhibitors (ChEIs), including donepezil, rivastigmine, and galantamine, as well as the N-methyl-D-aspartate antagonist, memantine, are approved for the symptomatic management of global cognitive impairment in AD [11]. Although those drugs improve cognitive performance, they are commonly associated with adverse events (AEs), such as drowsiness, loss of appetite, nausea, and vomiting [12]. Memantine, specifically, lacks evidence for efficacy in patients with mild AD [13]. There is also a lack of consensus among clinicians regarding issues related to initiation, optimal duration, and discontinuation [14]. The first disease modifying therapy, aducanumab, was recently approved by the FDA for AD, however, its approval is controversial due to ambiguous clinical trial results [15]. In patients with MCI, evidence does not support the use of ChEIs or memantine [16, 17], underscoring the need for effective treatments in this population in order to treat cognitive impairment in the early stages and help delay the progression from MCI to AD.

Individuals with AD frequently have neuropsychiatric symptoms (NPS). In a recent retrospective study, the most prevalent NPS among AD patients included apathy (51.9%) followed by irritability (41.0%) and depression (36.4%) [18]. Sleep disturbances (31.7%) were also commonly present in AD [18] and can have a negative impact on both quality of life and caregiver burden [19]. Current pharmacological treatments for NPS in dementia are only modestly effective and can be associated with significant adverse effects [20].

Transcranial direct current stimulation (tDCS) is a non-invasive brain stimulation technique that consists of applying a constant, low electric current between electrodes over the scalp in order to modulate cortical excitability [21]. Anodal tDCS, considered an excitatory stimulation, reduces the threshold required for neuronal firing and has been shown to improve neural efficiency, mood, and cognition in healthy as well as depressed and AD patients [22]. Similar to other types of brain stimulation, tDCS requires active neurons [23, 24], affecting the neurons that are closer to the discharge threshold (known as the activity-dependent model) [25, 26]. The effects of tDCS depend on the stimulation parameters used; however, studies have demonstrated that a single session of tDCS can induce changes in neuronal activity for up to 2 h [27]. tDCS has been used in numerous studies involving older, frail participants with no serious adverse events (SAEs) noted [28, 29]. In a review examining 158 studies, commonly reported side-effects included burning sensation, itching, tingling, headache, discomfort, dizziness, erythema, and fatigue. However, the risk of AEs did not increase in repeated sessions of active tDCS compared to sham tDCS [30].

To date, 21 studies have investigated the effects of repeated sessions of tDCS on cognitive performance in MCI and AD populations [31–51]. Some studies found that repeated sessions of tDCS significantly improved global cognition in mild AD patients [33, 52]. In addition, recall, recognition, and working memory have improved following single and repeated sessions of tDCS in mild AD [32, 53–58]. However, there was significant variability in treatment response which may be related to individual neurophysiological and biological status [59], including the level of cortical activity at the time of stimulations [60]. Based on new research on the activity-dependent model, studies have begun to investigate the use of combining other cognitive enhancing interventions with tDCS to prime neurons of interest [61]. In healthy participants, tDCS applied during a cognitive task resulted in greater improvement in performance of that cognitive task compared to when tDCS was applied at rest and when sham was applied during the task [62]. Although few studies have examined these combined therapies in AD patients, tDCS with cognitive training has been associated with greater improvements on some measures of cognition compared to sham tDCS with cognitive training, including the digit span, and trained and untrained picture-naming tasks [40].

Emerging evidence suggests that exercise may be an effective primer: a single bout of moderate-intensity aerobic exercise in healthy adults has been demonstrated to facilitate long-term potentiation-like neuroplasticity through increased motor-evoked potentials when exercise is performed prior to paired associative stimulation [27, 63]. Those findings were also associated with improved motor learning in participants who received exercise prior to stimulation compared to the rest condition (no exercise). In another study, a single bout of exercise (20 min of moderate-intensity cycling) resulted in increased cortical excitability and decreased intracortical inhibition compared to those who did not undergo an exercise session prior to stimulation [64]. Patients with Parkinson’s disease who received repeated sessions of tDCS with physical therapy demonstrated greater improvements in measures of verbal fluency compared to those who received sham and physical therapy [65]. A direct combination of tDCS and aerobic exercise may provide synergistic effects where aerobic exercise provokes large-scale changes across the brain, priming the brain for focal modulation, and leading to more robust effects [27]. In our previous study, the subgroup of participants who had cognitive impairment (baseline MoCA score ≤ 25) and who exercised at at least moderate-intensity level (≥70% of peak oxygen uptake (VO_2peak_)) significantly improved on the MoCA at 3 months [66]. Those results suggest that moderate-intensity exercise can improve global cognitive function in individuals who are cognitively impaired, and is consistent with exercise being an effective primer to enhance the cognitive benefits of tDCS.

In preclinical studies, tDCS has been shown to enhance secretion of brain-derived neurotrophic factor (BDNF), a neurotrophin with potent effects on neuronal survival and plasticity [67] and memory function [27, 68–71]. In vitro, application of direct current stimulation may also be associated with increases in angiogenic markers including vascular endothelial growth factor (VEGF) and angiopoietin-2 (ANGP-2) [72], which may play an important role in restoring memory through vascular survival [73]. In a vascular dementia mouse model, tDCS modulated oxidative stress through reduction of reactive oxygen species [74]. Reactive oxidative species can induce angiogenesis, and promote the production of angiogenic factors, such as VEGF [75]. These findings suggest that cognitive effects of tDCS may be partly mediated by neurogenesis and angiogenesis. In AD, peripheral BDNF concentrations are lower compared to controls [76]. In addition, vascular abnormalities leading to hypoperfusion are associated with increases in anti-angiogenic markers such as endostatin, which can contribute to AD pathology [77]. Although studies have demonstrated improved cognitive performance following tDCS in patients with MCI or AD [27, 60], there are no studies that have investigated angiogenic or neurogenic markers that may be mediating tDCS-induced cognitive improvement in MCI or AD patients. Given the negative impact of cognitive impairment in individuals with MCI and AD, this parallel-design study aims to determine whether a combination therapy with tDCS and exercise priming effectively improves cognitive function and NPS, and whether cognitive response to tDCS treatment is associated with markers of neurogenesis and angiogenesis.

### Objectives

The primary objective of the Exercise as a Primer for Excitatory Stimulation Study (EXPRESS) is to determine the efficacy of a combined exercise and tDCS intervention to enhance the effect of tDCS on global cognitive function.

Secondary objectives: to explore the efficacy of a combined exercise and tDCS intervention to enhance the effect of tDCS on specific cognitive domains and NPS.

Exploratory objectives: To identify mechanistic correlates of cognitive response to tDCS treatment through analysis of blood biomarkers.

## Methods

### Study population and eligibility criteria

Participants will be recruited through outpatient clinics within Sunnybrook Health Sciences Centre (SHSC) and referrals from physicians outside of SHSC within the Greater Toronto Area. Participants will be assessed based on inclusion and exclusion criteria highlighted in Table 1.

**Table 1:** List of inclusion and exclusion criteria

Inclusion	Exclusion
• Males or females ≥50 years of age (on day of randomization) • Clinical diagnosis of major or mild neurocognitive disorder due to AD or mixed AD/vascular disease following the DSM-5 [78] criteria by a psychiatrist • Mild severity of impairment (sMMSE score ≥ 19) [79] • Read and communicate in English	• Change in cognitive-enhancing medications (ChEIs and/or memantine) less than 3 months prior to study screen • Change in anticonvulsants or psychotropic medications less than 1 month prior to study screen • Currently taking benzodiazepines • Presence of metal implants that would preclude safe use of tDCS (e.g. pace-maker) • Significant neurological condition (e.g., epilepsy, Parkinson’s disease, multiple sclerosis) • Current psychiatric disorders (e.g. schizophrenia, bipolar disorder, depression, psychosis) or current substance abuse disorder • Medical contraindication to increasing physical activity level according to the Canadian Society of Exercise Physiology Questionnaire [80]

### Study design

**Fig. 1:**
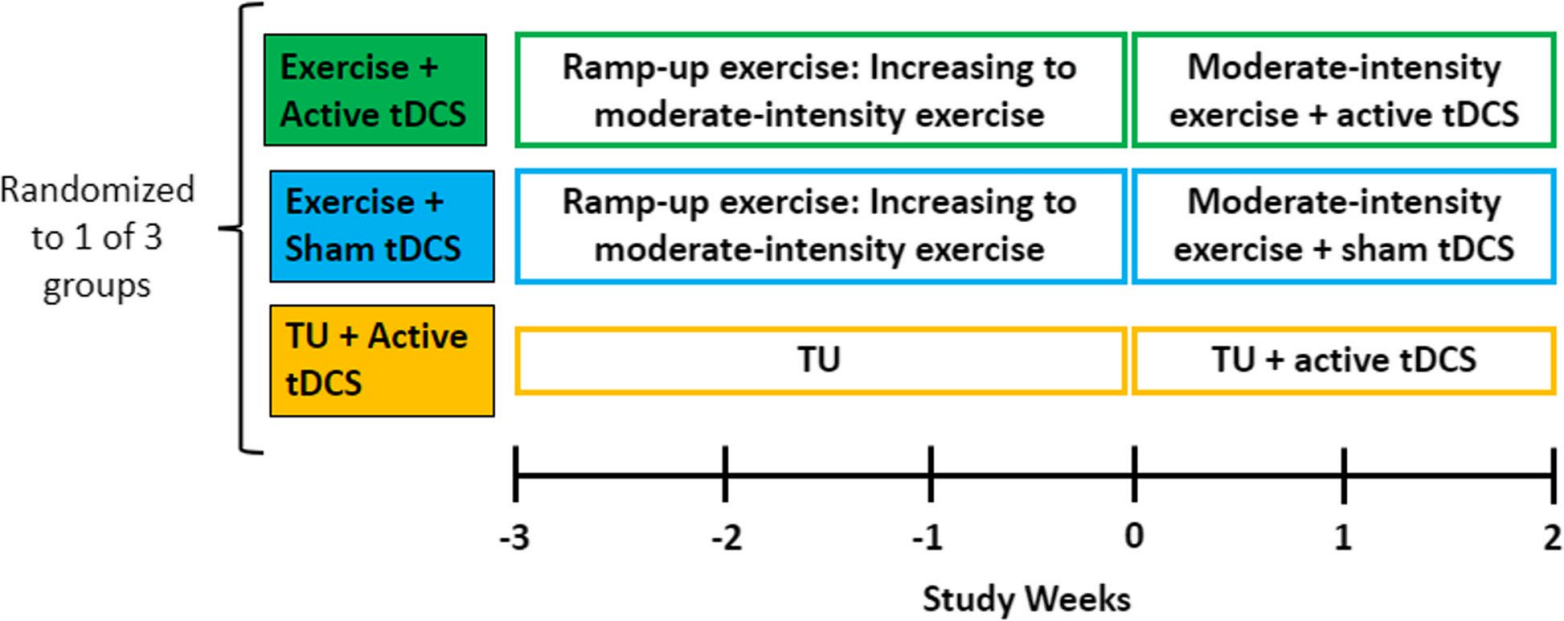
Study Design. Cognitive, NPS, and blood draw assessments are completed at the − 3, 0, and 2-week time points

This is a randomized, blinded, repeated-session, parallel-design study. Eligible participants will be randomized to one of three interventions: Exercise primer with tDCS, Treatment as usual (TU) with tDCS, or Exercise primer with sham tDCS (Fig. 1).

Assessments will be conducted at the following time points: Screening/Baseline (T1): within two weeks of beginning the 5-week study and/or on the first day of study initiation prior to any exercise intervention/TU, Midpoint (T2): 3 weeks into the study and prior to tDCS/sham, Endpoint (T3): study completion/at the end of the 5-week study, and Follow-Up with a physician (T4) (Table 2). At screening, inclusion and exclusion criteria (Table 1) will be assessed and demographic information (e.g. age, sex, ethnicity, education, sleep, caffeine, and alcohol intake, concomitant medications, comorbid illnesses, surgical and medical history, smoking status, as well as physical fitness level) and standardized Mini-Mental State Examination (sMMSE) score will be collected. At baseline, midpoint, and endpoint, cognitive and NPS outcomes, as well as blood biomarkers, will be collected. For participants randomized to receive an exercise intervention, an individual exercise prescription will be administered at baseline. For participants randomized to TU, written information in accordance with the Canadian Physical Activity Guidelines for older adults will be given at baseline. Exercise will be documented weekly using the Leisure-Time Exercise Questionnaire (LTEQ) for all participants to monitor exercise frequency and intensity throughout the study. Participants will be followed up by a qualified investigator (QI) or designated qualified physician within 2 weeks of study endpoint or termination.

**Table 2:** Schedule of assessments

	Screening/ Baseline	Midpoint	Endpoint^**a**^	Follow-up
Visit	T1 Study initiation	T2 3-weeks	T3 5-weeks / study endpoint	T4 Within 2 weeks of T3
***Demographics***				
Clinical diagnosis of mild or major NCD due to AD or mixed AD/vascular disease following the DSM-5 criteria by a psychiatrist	X			
Demographics	X			
Surgical and health history	X			
Comorbid Illnesses and concomitant medications	X	X	X	
Sleep, caffeine, alcohol intake, smoking status	X	X	X	
Standardized mini-mental state examination	X			
Canadian society of exercise physiology physical activity readiness questionnaire-plus	X			
***Outcomes***				
Montreal cognitive assessment	X	X	X	
Word recognition task	X	X	X	
Word recall task	X	X	X	
n-back	X	X	X	
Neuropsychiatric inventory	X	X	X	
Blood biomarkers	X	X	X	
**Safety**				
Adverse Events	X	X	X	
**Other**				
Leisure-time exercise questionnaire^b^	X	X	X	
Follow-up assessment with qualified investigator or designated qualified physician				X

^a^This visit will also take place if the patient withdraws from study or is terminated early

^b^The Leisure-Time Exercise Questionnaire will be administered weekly during the study

### Interventions

#### Exercise intervention

Current guidelines recommend that older adults participate in at least 150 min/week of moderate-intensity aerobic activity [81], a level sufficient to increase cortical excitability [64]. Following an initial cardiopulmonary exercise test at University Health Network-Toronto Rehabilitation Institute (UHN-TRI) to assess baseline cardiorespiratory fitness, an initial walking prescription will be given and set at a distance of approximately 0.8-1.6 km for 10-30 min depending on participant tolerance. The intensity will be at the ventilatory anaerobic threshold (VAT) and/or 60-80% of VO_2peak_. Prescriptions will be progressed every 3-4 sessions for 3 weeks to increase distance to reach a duration of 30 min and intensity to the level of the VAT and/or 80% of VO_2peak_. Participants will be instructed to exercise at UHN-TRI 3 times per week and at home 2 times per week for the first 3 weeks while the exercise intensity and/or duration is being increased. During the last 2 weeks, participants will be instructed to exercise at UHN-TRI 5 times per week. All exercise at UHN-TRI will be supervised and heart rate will be monitored using Polar heart rate monitors to ensure moderate intensity exercise is maintained. Prescribing intensity based on the VAT provides a metabolically uniform moderate intensity exercise prescription. This is especially important as not all individuals are able to reach a “true” physiological maximum on the exercise stress test. Our research group previously conducted a study in participants undergoing a 6-month cardiac rehabilitation exercise program at UHN-TRI, and demonstrated that exercise in older adults is a feasible intervention with 85.6% adherence [66].

#### Treatment as usual (TU)

TU will include routine advice about physical activity for older adults and written information in accordance with the Canadian Physical Activity Guidelines for older adults [82] as is standard of care. Physical activity in older adults often remains below the recommended levels of at least 150 min per week [83]. Participants in this group will not receive a personalized exercise plan and will not have contact from an exercise physiologist during the intervention. Participants will be instructed not to exercise before stimulation.

#### tDCS

**Fig. 2:**
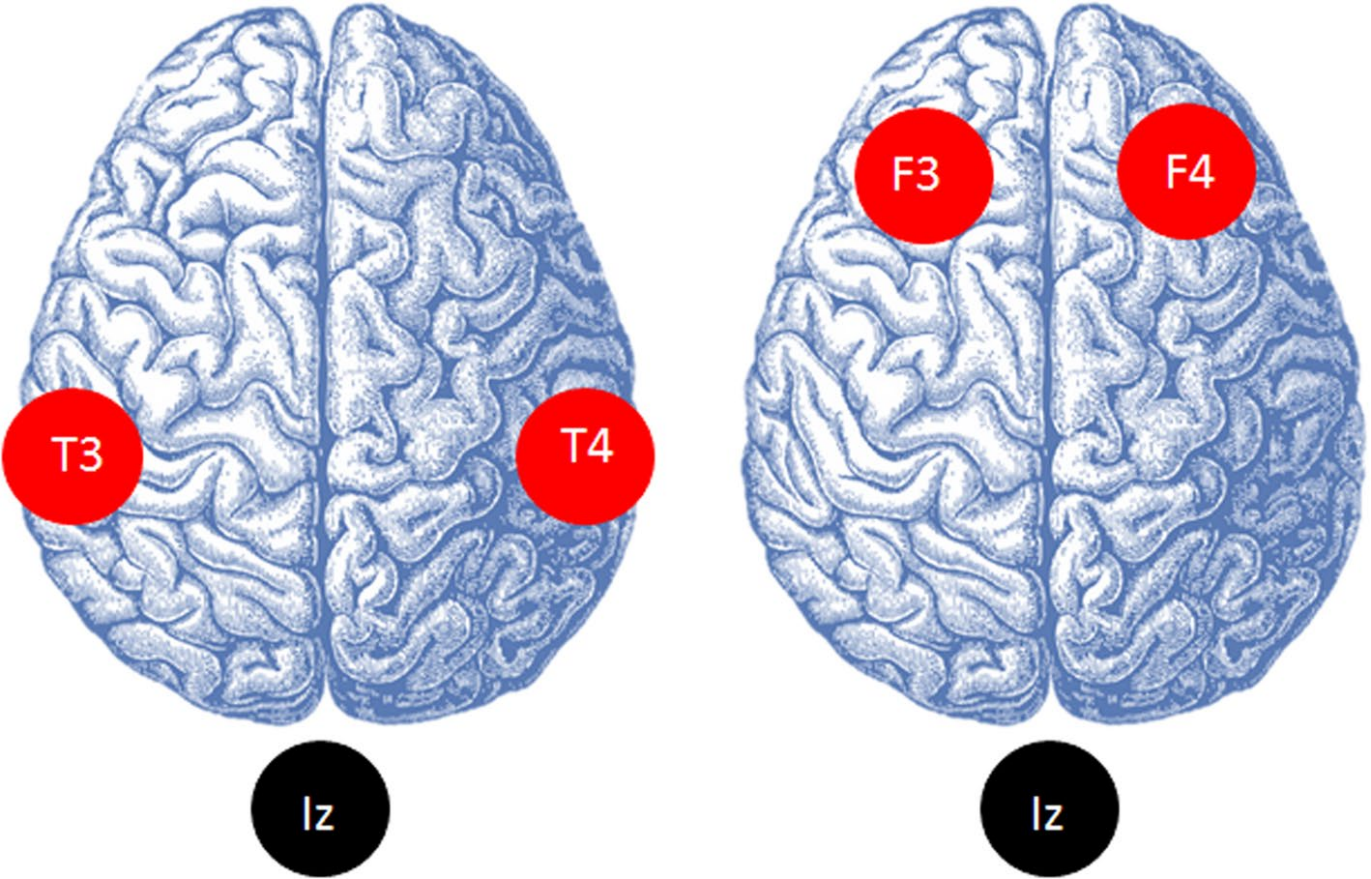
Schematic of electrode placements. Red and black circles represent anodal and return electrodes respectively

tDCS will be delivered by an Eldith DC stimulator (Magstim Company Ltd. UK), using three saline-soaked sponge electrodes held in place by an elastic band cap. The two anodal electrodes will be placed bitemporally over the left and right medial temporal lobes (T3 + T4 hemispheres) according to the International 10-20 system of electrode placement. Bitemporal placement with tDCS has shown cognitive improvements in MCI and AD [58], and neuroimaging findings on neuronal loss or hypoactivity in temporal regions of both hemispheres [84] also support the regions’ activity in memory processing. The return electrode will be placed at the inion (Iz) (Fig. 2). In each session, tDCS would be administered with a current strength of 2 mA for 20 min as these parameters are safe and commonly used in clinical research [85]. All study participants randomized to tDCS will receive tDCS 5 times/week for 2 weeks. Those randomized to receive exercise will be given tDCS following their exercise sessions. Compliance, including exercise and tDCS frequency, will be measured and documented.

#### Sham tDCS

The same procedure for tDCS will be used for the sham condition, except the 2 mA current will only be applied in the ramp-up phase (first 30 s). In previous studies [28, 86], this approach has ensured that the participants are blind to the type of stimulation (active versus sham).

### Study outcomes

#### Cognitive assessments

We will administer a comprehensive cognitive battery composed of neuropsychological tests that have been previously used in mild AD, and have demonstrated sensitivity to cognitive changes following tDCS [87]. Alternative versions will be used to avoid learning effects where applicable. The MoCA [88], a brief test of global cognition and a sensitive validated tool for detecting early cognitive changes in mild AD [89, 90], will be the primary outcome measure. The MoCA assesses multiple domains including orientation, attention, executive function, language and abstraction in addition to memory [90]. Two weeks is appropriate to observe changes in global cognition [33] following a cognitive enhancement intervention.

Working memory will be assessed using the n-back [91]. The Word Recognition Task and the Word Recall Tasks from the Alzheimer’s Disease Assessment Scale-Cog (ADAS-Cog) [92] will be used to assess recognition memory and recall memory respectively. These domains have also been shown to improve with exercise interventions [93, 94]. The sMMSE [79] will be administered at screening to assess for severity of cognitive impairment; it is the most common short screening tool for assessing cognitive impairment overall [95].

#### NPS

The Neuropsychiatric Inventory (NPI) [96] will be used to measure apathy, agitation, delusions, hallucinations, depression, euphoria, aberrant motor behaviour, irritability, disinhibition, anxiety, sleeping, and appetite disturbances in patients and to evaluate caregiver distress. Each domain is scored for frequency, severity and caregiver distress. Doman score is obtained by multiplying frequency and severity. Total NPI score is obtained by summing the individual domain scores. The NPI has good content validity, internal consistency, test-retest and interrater reliability, and is commonly used in research for the assessment of NPS in mild AD [97]. It will be administered to the patient’s caregiver through a standardized interview. Studies have shown that decreased cognition in MCI and AD is highly associated with increased NPS [98–100]. Therefore, effective treatments for cognitive impairment may also improve NPS in patients with AD.

#### Physical activity

The LTEQ [87] will be administered to the patient or the patient’s caregiver weekly to measure frequency and intensity of exercise completed. The Canadian Society of Exercise Physiology Questionnaire [80] will be administered to the patient or the patient’s caregiver at screening to assess for medical contraindications to a physical activity.

#### Biomarker assays

Angiogenic and neurogenic markers associated with exercise and tDCS outcomes, including BDNF, VEGF, ANGP-2, and endostatin, will be analysed using enzyme-linked immunosorbent assay [76, 101–103]. Samples for analyses will be frozen (− 80 °C) immediately and batched for analysis. These angiogenesis and neurogenic markers have previously been shown to change following two or more weeks of an exercise intervention [104–108].

### Randomization and blinding

A block randomization code will be computer-generated at SHSC and remain locked in a secure location in the department. The research personnel who will be administering the cognitive assessments will not be the interventionist administering the tDCS/sham intervention, and, with the remaining team members will be blinded to treatment allocation and block size until the final patient has completed follow-up and the database is locked. The patients will also be blinded to stimulation type. Unblinding will not be allowed unless there exist exceptional clinical circumstances that justify it (i.e., necessary for acute medical management of SAEs) and only after approval by the principal investigator (PI) or a QI.

### Safety profile and monitoring

Side-effects will be monitored using a customized checklist after each tDCS session. All emerging AEs that are clinically relevant based on a QI or designated qualified physician’s assessment will be noted and monitored until resolution. AEs will be appropriately described, i.e.: the association with the intervention will be coded as not related, possibly related or related; the determination of the severity and association will be decided by a QI. The QIs for this study will also be acting as the safety monitor, viewing all AEs.

New medical conditions developed during the study including musculoskeletal, cardiovascular or neurological symptoms which may make continued exercise unsafe will be reported to a QI or the designated qualified physician. Patients will be evaluated by a study physician to ensure that the participant received appropriate medical attention and to determine whether the participant may safely continue or discontinue the study.

An SAE is any untoward medical occurrence that results in death, is life-threatening (defined as an event in which the patient was at risk of death at the time of the event; it does not refer to an event which hypothetically might have caused death if it were more severe), results in persistent or significant disability or incapacity, requires hospitalization or causes prolongation of existing hospitalization, results in the development of drug dependency or drug abuse or is an important medical event defined as a medical event(s) that may not be immediately life-threatening or result in death or hospitalization but, based upon appropriate medical and scientific judgment, may jeopardize the patient or may require intervention (e.g., medical, surgical) to prevent one of the other serious outcomes listed in the definition above. SAEs will be collected during the study and will be followed until event resolution or stabilization. All SAEs that are unexpected and potentially related to the research will be reported in an expedited manner to the Research Ethics Board at SHSC.

### Adherence

Administration of exercise will be supervised by a physician, exercise physiologist, and designated research staff. Exercise frequency and intensity will be measured by the LTEQ weekly. Adherence to exercise regimen will be measured by information from the LTEQ and included as a covariate in statistical analyses.

### Statistical plan

#### Sample size calculation

The sample size calculation was performed using G*Power version 3.1.9.2 (Kiel, Germany). The sample size was based on findings from a study reporting a 2-point change in MMSE score following anodal tDCS in mild to moderate AD [33]. No studies to date have assessed changes in the MoCA following anodal tDCS. However, a strong correlation between the MMSE and MoCA (r = 0.86) in mild AD [89] implies that a similar change in the MoCA could be found in this population. Based on that assumption, a sample size of 60 completers (20 per treatment group) achieves 83% power for a repeated measures analysis of covariance (ANCOVA), adjusting for 4 covariates with an alpha of 0.05 to detect a medium effect size.

#### Statistical analyses

Multiple imputation methods will be used prior to analysis if missing data exceed 10%. A repeated measures ANCOVA will be used to assess between group differences in change in MoCA score over 2 weeks (treatment group x time interaction). Age and education will be added as covariates. A treatment group x time interaction will also be used to explore between-group differences in the n-back, ADAS-Cog, total NPI score, and NPI subscores. Repeated measures ANCOVAs will also be used to assess differences in markers of interest between treatment groups. Associations between changes in concentrations of BDNF, VEGF, ANGP-2, endostatin and MoCA scores over 2 weeks will be assessed using repeated measures linear regressions. It is expected that randomization will balance possible confounders.

### Data collection and management

Data will be collected on printed source documents/case report forms (CRFs). Source documents/CRFs include demographic information, medical information, cognitive as well as mood and behaviour assessments. Source documents, CRFs, and other records pertaining to the conduct of this study will be retained for 10 years, as per SHSC Research Ethics Board (REB) recommendations.

## Discussion

Recent studies have demonstrated that, individually, exercise and tDCS can enhance cortical excitability and improve cognitive functions in MCI and AD populations [60, 109]. Since tDCS may be most effective when applied to active neurons [23, 24], the combined treatment strategy, exercise-primed tDCS, may have greater efficacy to improve cognition in MCI and AD patients. To our knowledge, no studies have examined the cognitive response of exercise-primed tDCS in MCI or AD. The findings from this trial may provide a novel combined treatment strategy for MCI and mild AD patients.

A small number of studies have examined other combined interventions in MCI and AD, such as CT and tDCS [35, 40, 43, 44]; those studies only showed improvements in specific targeted cognitive domains, and it still remains unclear whether the combined treatment produces better outcomes than tDCS alone [110]. Exercise can induce large-scale changes across the brain, priming the brain for focal modulation, and leading to more robust effects with tDCS [27]. Also, exercise-primed tDCS is advantageous over other multimodal strategies, including exercise and transcranial magnetic stimulation, because it is easy to implement, time and cost-efficient, and safe with no-known severe side effects [27]. Recently, tDCS has been investigated as a home-based therapy for MCI patients [111].

Neurogenic factors, such as BDNF, have been suggested to have an influence on the response to tDCS [112]. Evidence has demonstrated that tDCS regulates BDNF expression and overall genetic variances of BDNF expression may be involved in the differences of individual responses to tDCS stimulation [71, 112]. Currently, there have been no studies in MCI or AD patients that have investigated neurogenic mechanisms of tDCS response. Findings on potential blood biomarkers associated with exercise, tDCS outcomes, angiogenesis and neurogenesis from this study may also identify potential mechanisms to delay impairment and preserve cognition in MCI and mild AD patients.

Given the potential wide availability of tDCS (available for home use) and current knowledge supporting efficacious exercise interventions, combined with the low risk of both interventions, this combination is highly feasible for widespread early intervention. As the populations suffering from MCI and AD are largely elderly individuals who are heavily burdened by pharmacological agents, the ability to introduce a non-pharmacological treatment is of great importance and value to patients and clinicians.

## Data Availability

The datasets generated during and/or analysed for the proposed study will not be publicly available as institutional approval would be required for data sharing. However, following publication of peer-reviewed journal articles, congregated data will be available from the corresponding author upon request.

## Abbreviations

AD: Alzheimer’s Disease
ADAS-Cog: Alzheimer’s Disease Assessment Scale-Cog
AEs: Adverse Events
ANCOVA: Analysis of Covariance
ANGP-2: Angiopoietin-2
BDNF: Brain-derived Neurotrophic Factor
ChEIs: Cholinesterase Inhibitors
CRFs: Case Report Forms
DSM-5: Diagnostic and Statistical Manual of Mental Disorders
EXPRESS: Exercise as a Primer for Excitatory Stimulation Study
Iz: Inion
LTEQ: Leisure-Time Exercise Questionnaire
MCI: Mild Cognitive Impairment
MoCA: Montreal Cognitive Assessment
NCD: Neurocognitive Disorder
NPI: Neuropsychiatric Inventory
NPS: Neuropsychiatric Symptoms
PI: Principal Investigator
QI: Qualified Investigator
REB: Research Ethics Board
SAEs: Serious Adverse Events
SHSC: Sunnybrook Health Sciences Centre
sMMSE: Standard Mini-Mental State Examination
TU: Treatment As Usual
tDCS: Transcranial Direct Current Stimulation
UHN-TRI: University Health Network-Toronto Rehabilitation Institute
VAT: Ventilatory Anaerobic Threshold
VEGF: Vascular Endothelial Growth Factor
VO_2peak_: Peak Oxygen Uptake
